# Production of Fatty Acid-Derived Valuable Chemicals in Synthetic Microbes

**DOI:** 10.3389/fbioe.2014.00078

**Published:** 2014-12-23

**Authors:** Ai-Qun Yu, Nina Kurniasih Pratomo Juwono, Susanna Su Jan Leong, Matthew Wook Chang

**Affiliations:** ^1^Department of Biochemistry, Yong Loo Lin School of Medicine, National University of Singapore, Singapore, Singapore; ^2^Synthetic Biology Research Program, National University of Singapore, Singapore, Singapore; ^3^Singapore Institute of Technology, Singapore, Singapore

**Keywords:** synthetic biology, metabolic engineering, fatty acid biosynthesis pathway, biochemical production, *Escherichia coli*, yeast

## Abstract

Fatty acid derivatives, such as hydroxy fatty acids, fatty alcohols, fatty acid methyl/ethyl esters, and fatty alka(e)nes, have a wide range of industrial applications including plastics, lubricants, and fuels. Currently, these chemicals are obtained mainly through chemical synthesis, which is complex and costly, and their availability from natural biological sources is extremely limited. Metabolic engineering of microorganisms has provided a platform for effective production of these valuable biochemicals. Notably, synthetic biology-based metabolic engineering strategies have been extensively applied to refactor microorganisms for improved biochemical production. Here, we reviewed: (i) the current status of metabolic engineering of microbes that produce fatty acid-derived valuable chemicals, and (ii) the recent progress of synthetic biology approaches that assist metabolic engineering, such as mRNA secondary structure engineering, sensor-regulator system, regulatable expression system, ultrasensitive input/output control system, and computer science-based design of complex gene circuits. Furthermore, key challenges and strategies were discussed. Finally, we concluded that synthetic biology provides useful metabolic engineering strategies for economically viable production of fatty acid-derived valuable chemicals in engineered microbes.

## Introduction

Fatty acids are one of the major components found in all organisms, usually in the intracellular forms of fatty acyl–acyl carrier protein (acyl-ACP), fatty acyl-coenzyme A ester (acyl-CoA), storage lipids, eicosanoids, and unesterified free fatty acids. In industry, applications of free fatty acids are generally limited due to the ionic nature of their carboxyl group (Peralta-Yahya et al., 2012). Comparatively, fatty acid derivatives have wider applications such as biofuels, biomaterials, and other biochemicals (Lennen and Pfleger, 2013; Runguphan and Keasling, 2014).

The low abundance or yield of fatty acid-derived chemicals in organisms renders their isolation from natural sources non-economically viable. The synthesis of fatty acid derivatives by chemical means also suffers from low efficiency and often requires harsh reaction conditions, prolonged times, and expensive equipment footprint (Song et al., 2013). The production of fatty acid-derived chemicals by engineering microbial cells into microbial factories is becoming an attractive alternative approach that can overcome the aforementioned bottlenecks associated with the other synthesis routes (Keasling and Chou, 2008; Schirmer et al., 2010; Lee et al., 2012).

To date, synthetic enzymatic pathways that lead to the production of fatty acid-derived valuable chemicals including fatty alkanes, fatty acid methyl/ethyl esters, fatty alcohols, hydroxy fatty acids, and lactones have been constructed in microorganisms such as *Escherichia coli* and *Saccharomyces cerevisiae*. However, it remains a challenge to achieve high yield, titer, and productivity of these fatty acid-derived chemicals. The major challenges faced in maximizing product titer are associated with: (1) improving the low enzyme activity of an entire metabolic pathway, (2) increasing the inadequate tolerance of the used microorganisms toward toxic target compounds, (3) recycling or replacing insufficient cofactors for enzymatic reactions, (4) enriching precursors and eliminating byproducts, and (5) optimizing and balancing the fluxes of whole metabolic networks to reduce burden on the host, and remove negative feedback regulation. Recently, advanced synthetic biology approaches have provided potential to address these challenging problems in re-engineering microbial systems for fatty acid-derived chemicals production (Clomburg and Gonzalez, 2010; Siddiqui et al., 2012; Zhang et al., 2012a), which narrows the gap toward realizing full-scale commercialization and industrialization of this manufacturing route.

In this review, we focus on the recent progress in metabolic engineering efforts to convert fatty acids to valuable chemicals using microbes as hosts, and advancement in synthetic biology approaches for further optimizing biochemical production in microbial biofactories.

## Metabolisms of Fatty Acids in Organisms

**Figure 1:**
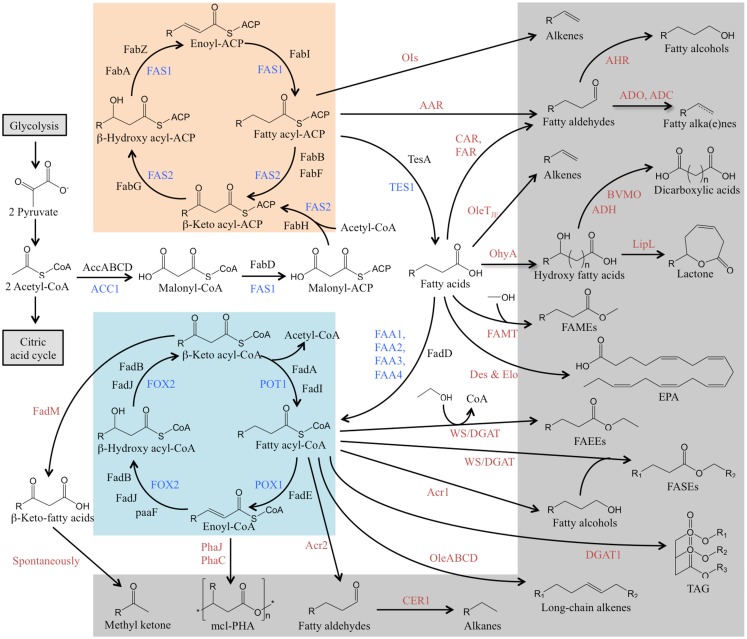
**Overview of metabolic pathways that lead to the production of fatty acids and fatty acid-derived chemicals**. The fatty acid biosynthesis (orange), β-oxidation cycle (blue), and the biosynthesis pathway of fatty acid-derived chemicals (gray) are presented. The enzymes of fatty acid metabolism in *S. cerevisiae* is in blue, in *E. coli* is in black, and the enzymes for conversion of fatty acids to their derivatives from other organisms is in red. AAR, acyl-ACP reductase; ACC1, acetyl-CoA carboxylase; AccABCD, a four subunits, biotin carboxyl carrier protein (AccB), biotin carboxylase (AccC), and acetyl-CoA carboxytransferase (AccA, AccD); Acr1 & Acr2, acyl-CoA reductase; ADC, aldehyde decarbonylase; ADH, alcohol dehydrogenase; ADO, aldehyde-deformylating oxygenase; AHR, aldehyde reductase; BVMO, Baeyer–Villiger mono-oxygenase; CAR, carboxylic acid reductase; CER1, fatty aldehyde decarbonylase Des, fatty acid desaturase; DGAT1, acyl-CoA:diacylglycerol acyltransferase; Elo, fatty acid elongase; FAA1 & FAA4, long-chain fatty acyl-CoA synthetase; FAA2 & FAA3, fatty acyl-CoA synthetase; FabA & FabZ, β-hydroxy acyl-ACP dehydratase; FabB, β-keto acyl-ACP synthase I; FabD, malonyl-CoA:ACP transacylase; FabF, β-keto acyl-ACP synthase II; FabG, β-keto acyl-ACP reductase; FabH, β-keto acyl-ACP synthase III; FabI, enoyl acyl-ACP reductase; FadA & FadI, β-keto acyl-CoA thiolase; FadB & FadJ, enoyl-CoA hydratase/β-hydroxy acyl-CoA dehydrogenase; FadD, fatty acyl-CoA synthase; FadE, acyl-CoA dehydrogenase; FadM, long-chain acyl-CoA thioesterase III; FAMT, fatty acid methyltransferase; FAR, fatty acid reductase; FAS1, acyl-CoA:ACP transferase/β-hydroxyl acyl-ACP dehydratase/acyl-ACP reductase; FAS2, acyl-ACP synthase/β-keto acyl-ACP synthase; FOX2, enoyl-CoA hydratase/β-hydroxyl acyl-CoA dehydrogenase; LipL, lactonizing lipase; OhyA, oleate hydratase; OleABCD, a four protein families for long-chain olefin biosynthesis; OleT_JE_, *Jeotgalicoccus* sp terminal olefin-forming fatty acid decarboxylase; OIs, a type I polyketide synthase for α-olefin biosynthesis; PaaF, 2,3-dehydroadipyl-CoA hydratase; PhaJ & PhaC, polyhydroxyalkanoate (PHA) synthases to yield medium-chain length polyester (mcl-PHA); POX1, fatty acyl-CoA oxidase; POT1, β-keto acyl-CoA thiolase; TE, acyl-ACP thioesterase; WS/DGAT, wax ester synthase/acyl-CoA:diacylglycerol acyltransferase.

Fatty acids are an integral part of all living organisms, and are generally composed of a hydrophobic hydrocarbon chain ending in one hydrophilic carboxylic acid functional group. The metabolic pathway of fatty acid metabolism in organisms is well-studied (Figure 1). Fatty acids are commonly built via *de novo* synthesis and elongation. Figure 1 shows that the *de novo* fatty acid synthesis starts from the primer acetyl-CoA and the extender malonyl-CoA through a cyclic series of reactions catalyzed by fatty acid synthases. The synthesized fatty acids are almost entirely composed of even-length and straight carbon chains that have various numbers of carbon atoms (<6, short chain; 6–12, medium chain; >14, long chain) and different degrees of unsaturation (saturated, monounsaturated, and polyunsaturated). Fatty acid breakdown takes place mainly via the β-oxidation pathway, which is like the *de novo* synthesis pathway running in a reverse direction (Figure 1).

The fatty acid metabolic pathway generates both fatty acids and their derivatives. The fatty acids and their derivatives from the synthesis and breakdown pathways can ultimately be converted to desirable value-added chemicals through metabolic engineering.

## Metabolic Engineering

Metabolic engineering is undoubtedly an essential tool in biocatalytic systems because it can develop new cell factories or improve existing cell factories to produce non-native compounds. The primary objective of metabolic engineering is to improve the cellular properties by intentional modification of organisms through redirecting metabolic fluxes. Traditionally, metabolic engineering is performed by introducing completely new pathways for production of novel proteins, drugs, chemicals, or modifying native pathways to achieve desired metabolic goals such as high productivity of metabolites and high robustness of host strains. Here, metabolic engineering relies on directed genetic perturbations, usually in terms of modifying the promoter activity of a given gene, performing over-expression or deletion of endogenous genes/enzymes/pathways, and utilizing heterologous expression of genes/enzymes/pathways (Ostergaard et al., 2000).

However, traditional metabolic engineering approaches frequently fail to lead to the desired phenotypes because of unclear or complex gene structures, functions, and regulations in cellular metabolic networks. Hence, more efforts are required to achieve an integrative and holistic view of the overall network of pathways in organisms rather than individual pathways, which can then guide rational design strategies.

It is challenging to reconstruct certain biochemical pathways in a dynamic metabolic network without having the entire information on intracellular gene regulatory, metabolic, and signaling networks. Thus, fundamental knowledge on cellular genetics, biochemistry, and physiology is critical. Recently, multiple analytical and modeling tools, such as genomics, transcriptomics, proteomics, metabolomics, fluxomics, high-throughput screening, and *in silico* studies, have been utilized to elucidate metabolic engineering workflows, which provide useful information to predict the altered behaviors of metabolic networks, guide strain design, and maximize the efficacy of metabolic engineering.

## Microbial Hosts for the Production of Fatty Acid-Derived Chemicals

Metabolic engineering of microbial systems provides a renewable route to produce desired organic molecules such as fuels, materials, and chemicals. Many different types of microbes can naturally produce and accumulate varying levels of fatty acids efficiently. Some of them exhibit properties advantageous to the production of fatty acid-derived compounds through metabolic engineering.

*Escherichia coli* and *S. cerevisiae* are the most intensively studied and widely used model microorganisms in the development of metabolic engineering strategies aimed at providing heterologous bioproduction of value-added metabolites. They have several key advantages such as lower safety risks, faster growth rates, good tractability, more well-studied, and more industrially relevant. So far, a number of fatty acid-derived chemicals have been successfully produced in metabolically engineered *E. coli* and *S. cerevisiae* (for references, see Table 1 below). Compared to *E. coli*, *S. cerevisiae* can be cultured at higher cell density and has a better fermentation performance at low temperature and pH (Aronsson and Ronner, 2001; Ageitos et al., 2011). *S. cerevisiae* is also more suited for the functional expression of eukaryotic enzymes (many enzymes involved in fatty acid production are from the plant kingdom) due to its endomembrane systems and post-translational modifications (Ageitos et al., 2011). However, in many cases, the production yields of fatty acid-derived chemicals from the engineered *S. cerevisiae* are much lower than those of *E. coli* when overexpressing identical heterologous genes. The reasons for this are not clearly understood.

**Table 1:** **Examples of valuable fatty acid-derived chemicals produced by metabolically engineered microorganisms**.

Chemical	Organism	Titer	Reference
FAMEs (C12–18)	*E. coli*	16 mg/L	Nawabi et al. (2011)
FAEEs (C12–20)	*E. coli*	1.5 g/L	Zhang et al. (2012a)
	*S. cerevisiae*	47.6 mg/L	Shi et al. (2014)
FASEs (C12–18)	*E. coli*	1.05 g/L	Guo et al. (2014)
Butanol	*E. coli*	0.81 mg/L	Mattam and Yazdani (2013)
	*E. coli*	30 g/L	Shen et al. (2011)
	*S. cerevisiae*	2.5 mg/L	Steen et al. (2008)
	*S. cerevisiae*	242.8 mg/L	Si et al. (2014)
	*C. saccharoperbutylacetonicum*	32.8 g/L	Richter et al. (2012)
3-Methyl-1-pentanol	*E. coli*	384.3 mg/L	Zhang et al. (2008)
Fatty alcohols (C4–5)	*E. coli*	4035 mg/L	Huo et al. (2011)
Fatty alcohols (C6–10)	*E. coli*	0.33 g/L	Dellomonaco et al. (2011)
Fatty alcohols (C12, C14)	*E. coli*	0.45 g/L	Zheng et al. (2012)
Fatty alcohols (C12–18)	*E. coli*	1.725 g/L	Liu et al. (2013)
Fatty alcohols (C16–18)	*E. coli*	0.1 g/L	Zheng et al. (2012)
	*S. cerevisiae*	0.1 g/L	Runguphan and Keasling (2014)
Pentane	*Y. lipolytica*	4.98 mg/L	Blazeck et al. (2013)
Alkanes	*E. coli*	580.8 mg/L	Choi and Lee (2013)
*Iso-*alkanes	*E. coli*	5 mg/L	Howard et al. (2013)
Alkanes/Alkenes	*E. coli*	300 mg/L	Schirmer et al. (2010)
Alkenes	*E. coli*	97.6 mg/L	Liu et al. (2014)
Long-chain alkenes	*E. coli*	40 μg/L	Beller et al. (2010)
Hydroxy fatty acid (C18)	*S. pombe*	137 μg/L	Holic et al. (2012)
Hydroxy fatty acid (C18)	*Y. lipolytica*	60 mg/g DCW	Beopoulos et al. (2014)
Hydroxy fatty acid (C14)	*C. tropicalis*	174 g/L	Lu et al. (2010)
ω-1-Hydroxy fatty acid	*B. pumilus*	570 mg/L	Dellomonaco et al. (2011)
Dicarboxylic acid (C14)	*C. tropicalis*	210 g/L	Picataggio et al. (1992)
Methyl ketone	*E. coli*	500 mg/L	Park et al. (2012)
Lactone	*Y. lipolytica*	11 g/L	Wache et al. (2003)
ω-3-eicosapentaenoic acid (EPA)	*Y. lipolytica*	0.15 g/g DCW	Xue et al. (2013)
Triacylglyceride (TAG)	*E. coli*	1.1 mg/L	Rucker et al. (2013)
Poly-3-hydroxylalkanoates (mcl-PHA)	*E. coli*	0.4 g/L	Wang et al. (2012)
Medium-chain fatty acids (MCFAs)	*E. coli*	263 mg/L	Torella et al. (2013)

*DCW, dry cell weight*.

Oleaginous microorganisms, which include bacteria, yeast, cyanobacteria, microalgae, and filamentous fungi, can accumulate intracellular lipids to at least 20% of their cellular dry mass. Thus they are considered attractive next-generation host candidates for production of fatty acid-derived chemicals because these oleaginous species have the ability to provide fatty acids or lipids as precursors (Ratledge, 1994). Oleaginous bacteria have been less studied to date because the lipid content in oleaginous bacteria is relatively lower than that in yeast, cyanobacteria, microalgae, and filamentous fungi, and they are also limited by lower growth rates. Oleaginous cyanobacteria and microalgae are attractive hosts for fatty acid-derived chemical production mainly because of their unique photosynthesis capability that directly converts solar energy and recycles CO_2_ into fuels (Parmar et al., 2011). For instance, cyanobacteria *Synechococcus elongatus* sp. strain PCC 7942 have already been successfully engineered to produce a number of different biofuel related compounds, including 1-butanol (Lan and Liao, 2012), isobutanol (Atsumi et al., 2009), isobutyraldehyde (Atsumi et al., 2009), and 2-methyl-1-butanol (Shen and Liao, 2012). However, they are both technically difficult to manipulate genetically, and their cultivation and growth processes are more complicated and expensive than bacteria, yeast, and fungi. These hurdles have hampered their use in the production of fatty acid-derived chemicals through metabolic engineering. Similarly, the exploitation of oleaginous filamentous fungi as production hosts is also impeded by the lack of efficient genetic transformation techniques.

In comparison, oleaginous yeast has many advantages over other oleaginous microbial sources that makes this class of microbes the most promising cell factories for the production of fatty acid-derived chemicals. They can grow to high cell densities in simple and inexpensive culture, reaching extremely high levels of lipid accumulation of more than 70% of their dry weight (Beopoulos et al., 2008; Santamauro et al., 2014). They are also able to use different kinds of residues in waste resources as nutrients (Papanikolaou et al., 2003; Fickers et al., 2005). They are more genetically tractable than oleaginous cyanobacteria, microalgae, and filamentous fungi with relatively well-developed genetic tools (Madzak et al., 2004). Oleaginous yeast candidates, which show great potential as hosts for fatty acid-derived chemical production, include *Yarrowia lipolytica* (Blazeck et al., 2014), *Lipomyces starkeyi* (Tapia et al., 2012), *Lipomyces tetrasporus* (Lomascolo et al., 1994), *Rhodotorula glutinis* (Saenge et al., 2011), *Rhodosporidium toruloides* (Li et al., 2007), *Cryptococcus albidus* (Fei et al., 2011), *Cryptococcus curvatus* (Gong et al., 2014), *Metschnikowia pulcherrima* (Santamauro et al., 2014), *Trichosporon pullulans* (Huang et al., 2011), and *Waltomyces lipofer* (Raschke and Knorr, 2009). In particular, the model oleaginous yeast *Y. lipolytica* provides a promising platform as an oleaginous cell factory to convert fatty acids to more valuable metabolites. This oleaginous platform has the ability to utilize wide-scale renewable materials as substrates (Papanikolaou et al., 2003; Fickers et al., 2005) and multiple cheap carbon sources for growth (Papanikolaou et al., 2002; Athenstaedt et al., 2006). Furthermore, it is more competitive than the non-oleaginous yeast *S. cerevisiae* in terms of lipid yield and heterologous protein yield (Gellissen et al., 2005; Papanikolaou and Aggelis, 2009). All of these features make *Y. lipolytica* very attractive for use in the production of fatty acid-derived products. Recently, production of various fatty acid-derived biofuel and bioproducts using engineered *Y. lipolytica* has been investigated, including compounds such as triglycerides (Tai and Stephanopoulos, 2013), alkanes (Blazeck et al., 2013), lactones (Wache et al., 2003), hydroxy fatty acids (Beopoulos et al., 2014), dicarboxylic acids (Wache, 2013), and polyunsaturated fatty acids (Xue et al., 2013). However, transport mechanisms, transcriptional regulatory, and signal transduction pathways involved in lipid accumulation and degradation in *Y. lipolytica* need further exploration. This will pave the way to better utilization of this platform.

## Metabolic Engineering of Microbes for Producing Fatty Acid-Derived Chemicals

As discussed above, most fatty acid-derived chemicals are hard to obtain efficiently from natural sources or through native metabolic pathways. Recent efforts of metabolic engineering have been made in developing microbial chemical factories for the production of target chemicals. Figure 1 shows that the chemicals derived from fatty acids are generated by introducing the corresponding conversion steps associated with native fatty acid metabolic pathways. In this section, we describe pathway engineering for biochemical synthesis and review applications of metabolic engineering in the production of various fatty acid-derived chemicals, including: (1) fatty acid esters; (2) fatty alkanes and alkenes; and (3) fatty alcohols and other chemicals such as fatty ketones and lactones.

### Metabolic engineering toward fatty acid ester production

Fatty acid methyl esters (FAMEs) and fatty acid ethyl esters (FAEEs) can be used as “biodiesel” fuel. The key enzyme to synthesize FAEEs in engineered microbes is wax ester synthase, which is responsible for catalyzing the esterification reaction of acyl-CoAs and alcohols.

In *S. cerevisiae*, by expressing heterologous wax ester synthase from *Marinobacter hydrocarbonoclasticus* DSM 8798 and up-regulating endogenous acetyl-CoA carboxylase, FAEEs were produced at a final titer of 8.2 mg/L (Shi et al., 2012). By further eliminating pathways for triacylglycerols (TAG) formation, steryl esters (SE) formation, and β-oxidation that compete with FAEE forming pathway, the production of FAEEs was at 17.2 mg/L in the strain lacking these non-essential fatty acid utilization pathways (Valle-Rodriguez et al., 2014). The corresponding FAEE production increased up to 34 mg/L after integrating the wax ester synthase gene cassette into the yeast genome. To further improve FAEE production, endogenous acyl-CoA binding protein, and NADP^+^-dependent glyceraldehyde-3-phosphate dehydrogenase from *Streptococcus mutans* were overexpressed in the final integration strain. The highest FAEE titer of 47.6 mg/L was achieved (Shi et al., 2014). In *E. coli*, FAEEs at 674 mg/L were produced by using combinatorial approaches: (1) over-expression of wax ester synthases from *Acinetobacter baylyi* for conversion of fatty acids to FAEEs, native acyl-ACP thioesterases and acyl-CoA ligases for acyl-CoA production, pyruvate decarboxylase and alcohol dehydrogenase from *Zymomonas mobilis* for non-native ethanol-forming, and (2) deletion of the competing fatty acid β-oxidation pathway (knockouts are *fadE*) (Steen et al., 2010). It was reported that over-expression of acetyl-CoA carboxylase and optimization of cultivation conditions further improved the yield of FAEEs to 922 mg/L (Duan et al., 2011). A recent work demonstrated that a dynamic sensor-regulator system increased the FAEEs titer to 1.5 g/L in genetically engineered *E. coli* strain (Zhang et al., 2012a). Fed-batch pilot scale cultivation of the engineered *E. coli* p(Microdiesel) strain could yield 15 g/L FAEEs, by first using glycerol as sole carbon source for biomass production before glucose and oleic acid were added as carbon sources (Elbahloul and Steinbüchel, 2010).

In *E. coli*, FAMEs were formed from free fatty acids and S-adenosylmethionine through expressing fatty acid methyltransferases from *Mycobacterium marinum* and *Mycobacterium smegmatis*. Over-expression of heterologous thioesterases can increase free fatty acids, and further result in increased FAME synthesis. It was reported that over-expression of thioesterases such as thioesterase II from *E. coli*, acyl-ACP thioesterases from *Clostridium phytofermentans*, *Clostridium sporogenes*, *Clostridium tetani* and *M. marinum*, 3-hydroxyacyl ACP:CoA transacylases from *Pseudomonas putida*, and methionine adenosyltransferases from rat, combined with deletion of a global methionine regulator *metJ*, led to the production of FAMEs at up to 16 mg/L (Nawabi et al., 2011).

### Metabolic engineering toward fatty alka(e)ne production

Fatty alka(e)nes can exist as straight or branched chains. Both straight- and branched-chain alka(e)nes have the potential to serve as advanced biofuels. There are two primary pathways for alka(e)ne biosynthesis: (1) a pathway that starts from acyl-ACP, followed by reducing acyl-ACPs to form fatty aldehydes catalyzed by reductases, and then converting fatty aldehydes to alka(e)nes by aldehyde decarbonylases; and (2) a pathway that starts from free fatty acids, followed by reduction and decarboxylation to generate alka(e)nes.

Over-expression of acyl-ACP reductases and aldehyde decarbonylases from cyanobacteria in *E. coli* and *Synechocystis* sp. PCC 7002 achieved alka(e)ne concentration at 300 mg/L (Schirmer et al., 2010) and 5% of cell dry weight (Reppas et al., 2010), respectively. Recently, in *E. coli*, free fatty acids were catalyzed to form fatty aldehydes by expressing fatty acid reductase complex from *Photorhabdus luminescen*. Coupled with aldehyde decarbonylases from *Nostoc punctiforme*, fatty aldehydes were converted further to alka(e)nes. In this study, production of branched-chain alka(e)nes from branched-chain fatty acids at a titer of 2–5 mg/L was also reported by over-expression of branched-chain α-keto acid dehydrogenase complex and β-ketoacyl-ACP synthase III from *B. subtilis* (Howard et al., 2013).

Terminal alkenes can also be produced in microorganisms via two pathways: (1) conversion of free fatty acids to terminal alkenes by cytochrome P450 peroxygenase (Rude et al., 2011); and (2) conversion of acyl-ACP to terminal alkenes by a large multi-domain type I polyketide synthases (Mendez-Perez et al., 2011). However, the pathways involving free fatty acids and acyl-ACP need to be further optimized to improve the efficiency and yield. Very long-chain alkenes can be generated by a head-to-head condensation of two acyl-CoAs catalyzed by the OleABCD protein families. In a previous study, heterologous expression of the Ole cluster from *Micrococcus luteus* ATCC 4698 in *E. coli* led to the production of very long-chain alkenes at a total concentration of 40 μg/L (Beller et al., 2010).

### Metabolic engineering toward production of fatty alcohols and other chemicals

Fatty alcohols (or long-chain alcohols) can be formed by reduction from fatty aldehyde intermediates using aldehyde reductases, for example, from cyanobacterium *Synechocystis* sp. PCC 680 (Steen et al., 2010). Fatty alcohols can also be directly produced by acyl-CoA reductases from *M. aquaeolei*, mouse, jojoba, and *Arabidopsis thaliana*. Another fatty aldehyde reductase from *M. aquaeolei* was found to possess the ability to catalyze not only fatty aldehydes but also acyl-CoA or acyl-ACP to corresponding fatty alcohols (Hofvander et al., 2011; Liu et al., 2013). In these pathways, fatty aldehyde intermediates can be bypassed (Tan et al., 2011). In addition, another synthetic pathway leading to 1-butanol (short-chain fatty alcohol) production from *Clostridium* species was functionally constructed in *E. coli* (Shen et al., 2011), *S. cerevisiae* (Steen et al., 2008), and *Thermoanaerobacterium saccharolyticum* (Bhandiwad et al., 2014). This pathway begins with a CoA-dependent Claisen condensation reaction of two acetyl-CoA followed by reduction, dehydration, and hydrogenation. Thus, this sequence of chemical reactions is the reverse direction of that in β-oxidation pathway. Recently, this CoA-dependent 1-butanol synthesis pathway has been extended to produce other linear short-chain fatty alcohols (C6–C8) in *E. coli* (Zhang et al., 2008; Tseng and Prather, 2012).

In addition, chemicals derived from fatty acids also include methyl ketones, hydroxy fatty acids, lactones, and dicarboxylic acids. Methyl ketones can be synthesized through conversion of fatty acids to β-keto acyl-CoAs in β-oxidation, and hydrolysis of β-keto acyl-CoAs by thioesterases to form β-keto fatty acids, followed by decarboxylation of β-keto fatty acids to methyl ketones (Goh et al., 2012). Hydroxy fatty acids can be synthesized by diverse kinds of fatty acid-hydroxylation enzymes, including P450, lipoxygenase, hydratase, 12-hydroxylase, and diol synthase (Kim and Oh, 2013). Lactones can be generally obtained by one-step biotransformation of the precursors hydroxy fatty acids (Wache et al., 2003). To generate dicarboxylic acids, hydroxy fatty acids can be oxidized to fatty ketones by alcohol dehydrogenases, followed by further oxidation of the fatty ketones to esters by Baeyer–Villiger monooxygenases. The esters are subsequently hydrolyzed by esterases to yield dicarboxylic acids (Song et al., 2013). The representatives of valuable chemicals derived from fatty acids in engineered microbes are listed in Table 1.

Taken together, metabolic engineering of microorganisms serves as a good platform for effective production of desired fatty acid-derived valuable chemicals. However, more research efforts are required to achieve industrially relevant titers of these chemicals.

## Facilitation of Fatty Acid-Derived Chemical Bioproduction with Advanced Synthetic Biology Tools

Successful production of fatty acid-derived chemicals by metabolic engineering of microbial systems has already been achieved. However, the productivity and titers of each of these processes remain to be improved. Further improvement in production efficiency is critical because high productivity and product yield for cost-effective production are the most important pre-requisites for large-scale industrial production of fatty acid-derived chemicals that is also financially viable.

Recent years have witnessed the emergence and marked progress in synthetic biology. Many advanced synthetic biology tools have offered a variety of applications to improve the ability to re-engineer microbial cells for achieving high yields of valuable chemicals, e.g., modular control over metabolic flux in mevalonate biosynthesis pathway using synthetic protein scaffolds in *E. coli* (Dueber et al., 2009), enhancement in production of fatty acid-derived biofuels by using dynamic sensor-regulator system in *E. coli* (Zhang et al., 2012a) and improvement of tolerance against alkane biofuels by transporter engineering in *S. cerevisiae* (Chen et al., 2013a). Although these tools are not widely used in metabolic engineering of microorganisms aiming to produce fatty acid-derived chemicals, there is no doubt that these innovations would facilitate tremendous potential for improved metabolic engineering of microbial systems in the production of various fatty acid-derived products.

**Figure 2:**
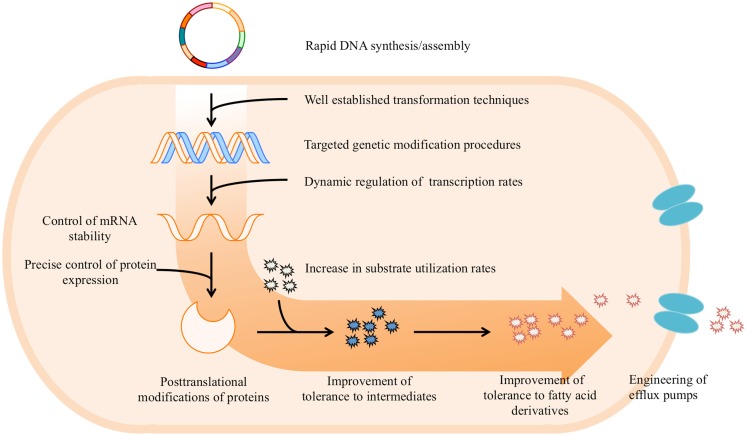
**Overview of potential applications of synthetic biology tools for construction and optimization of metabolic pathways that increase production of fatty acid-derived chemicals**.

In summary, advanced synthetic biology approaches for pathway optimization show great promise in enhancing the speed and efficiency of creating improved microbial strains in combination with common metabolic engineering efforts. The production of fatty acid-derived chemicals could benefit from the integration of synthetic biology tools with the work already accomplished through metabolic engineering. Thus implementation of advanced synthetic biology tools in redesigning fatty acid biosynthesis pathway and heterologous metabolic pathways for the production of fatty acid-derived targets will guide rational manipulation for production of our target at high yields and titers. In this section, we will briefly review the recent development of synthetic biology methodologies and possible applications for construction and optimization of metabolic pathways in microbes at DNA, transcription, translation, and post-translation levels (Figure 2).

### DNA engineering

The first step of most metabolic engineering and synthetic biology studies is to reconstruct a completely or partially synthetic pathway. Therefore, rapid assembly of heterologous pathways with many enzymatic steps is a major challenge in metabolic engineering. Traditional DNA molecular cloning approaches, which are tedious, time-consuming and mainly limited by template-based synthesis, restriction digestion, and ligation-based cloning, are increasingly being replaced with *de novo* DNA synthesis and more sophisticated assembly capabilities. Many simple, rapid, high-throughput, high-fidelity and low-cost DNA synthesis, and assembly methods in synthetic biology have been developed, including programmable microfluidic chips (Tian et al., 2004), BioBricks assembly (Sleight et al., 2010), BglBricks assembly (Anderson et al., 2010), In-Fusion assembly (Zhu et al., 2007), Gibson DNA assembly (Gibson et al., 2009), TAR-based assembly (Benders et al., 2010), Circular polymerase extension cloning (CPEC) (Quan and Tian, 2009), Sequence and ligase independent cloning (SLIC) (Li and Elledge, 2007), Seamless Ligation Cloning Extract (SLiCE) (Zhang et al., 2012c), DNA assembler (Shao et al., 2009), Uracil-specific excision reagent cloning (USER) (Gulig et al., 2009), Methylation-assisted tailorable ends rational ligation (MASTER) (Chen et al., 2013b), Site-specific recombination-based tandem assembly (SSRTA) (Zhang et al., 2011), PCR-based two-step DNA synthesis (PTDS) (Xiong et al., 2004), Golden Gate assembly (Cermak et al., 2011), and Polymerase incomplete primer extension cloning (PIPE) (Liu and Naismith, 2008). These approaches together enable the efficient synthesis of synthetic DNA fragments with no apparent limits on either sequence or length. Therefore, these powerful and efficient toolboxes allow efficient manufacture of genes, regulatory elements, circuits, gene clusters, and metabolic pathways for the production of novel chemicals.

The laborious and site-specific gene targeting by homologous recombination techniques, which have limited applicability for genome wide modification are now being increasingly displaced with such genome-scale engineering techniques as multiplex automated genome engineering (MAGE), conjugative assembly of genome engineering (CAGE), and transcription activator-like effector nucleases (TALENs). MAGE simultaneously targets multiple locations on chromosomes to introduce small modifications in a single cell or across a population of cells, facilitating rapid generation of a diverse set of genetic changes. 1-deoxy-d-xylulose-5-phosphate (DXP) biosynthesis pathway in *E. coli* was optimized by this technique. Twenty-four genetic components in the DXP pathway were modified simultaneously using a complex pool of synthetic oligonucleotides, creating over 4.3 billion combinatorial genomic variants per day and achieving a more than fivefold increase in lycopene production within 3 days (Wang et al., 2009). CAGE enabled large-scale assembly of many modified genomes on the basis of MAGE (Isaacs et al., 2011). TALENs is another powerful tool created to target double-strand breaks at specific locations in the genome (Christian et al., 2010).

### Transcriptional engineering

Transcription is the first dedicated phase of gene expression and therefore, different toolsets have been developed in synthetic biology for controlling gene expression and modulating RNA levels in the engineered cells. The primary goal of transcriptional engineering is synthetic control of RNA transcription and transcript levels by controlling gene copy number, transcription initiation rate, transcription termination efficiency, and transcript decay rate. Modifications of gene copy number can be achieved by changing the origin of replication of recombinant expression plasmids or the number of chromosomally integrated gene copies (in particular, strategies for chromosomal integrations at multiple loci). In addition, promoter engineering can be applied to regulate the rate of transcription initiation by using different types of promoters such as constitutive promoters, inducible promoters, specific promoters, hybrid promoters, synthetic promoters, and synthetic promoter libraries (De Mey et al., 2007). Transcription termination efficiency can be regulated as well by changing terminator sequence contexts (Cambray et al., 2013). Studies on mRNA folding and degradation rates determined by mRNA message itself (primary sequences and/or secondary-structures) and on the genomic region of 5′- and 3′-UTR allowed for further control of transcript abundances of genes of interest (Dori-Bachash et al., 2011; Zaborske et al., 2013).

Based on the principles above, increasing attempts have been recently made to further improve the sensitivity and precision of transcription regulation. First, RNA control system by engineered RNA hairpins enables conditional activation of an endogenous pathway capable of operating in autonomous mode within a complex cellular regulatory network (Venkataraman et al., 2010). Second, dynamic sensor-regulator system uses a transcription factor to specifically sense key intermediates and dynamically regulates the expression of genes. In biodiesel biosynthetic pathways in *E. coli*, this system substantially improved the stability of biodiesel-producing strains and increased the yield by threefold. This strategy can also be extended to other biosynthetic pathways to balance metabolism, thereby increasing product titers and conversion yields and stabilizing production hosts (Zhang et al., 2012a). Third, regulatable expression system has been developed for modulating gene expression in *Corynebacterium glutamicum*. Furthermore, this work provided a synthetic promoter library that enabled the selection of strong promoters. This technology should have many future applications for optimizing bioproduction in *C. glutamicum* and other organisms (Rytter et al., 2014). In addition, transcription factor engineering (Lee et al., 2011) and global transcription machinery engineering study (Zhang et al., 2012b) also serve as a good example for using synthetic biology tools to re-engineer transcriptional regulation in organisms. All these efforts have already shown promise and could lead to highly optimized expression of synthetic pathways at the transcriptional level.

### Translational engineering

After gene transcription is complete, translational engineering tools can be used to speed translation rates, lower degradation rates, and tune protein yields. Synthetic ribosome binding sites (Salis, 2011), antisense RNA (Chang et al., 2012), ribozymes (Meaux and Van Hoof, 2006), translation machinery (rRNA, tRNA, and amino acid) (Harris and Jewett, 2012), peptide tags, and codon optimization method have been proved effective in control of cellular protein levels at the translational level. mRNA secondary structure engineering is a newly developed method for translational regulation of gene expression. The engineered mRNA molecules that exhibit diverse activities including sensing, regulatory, information processing, and scaffolding activities has been implemented as key control elements in synthetic genetic networks to program biological function (Liang et al., 2011). Compared with DNA engineering and transcriptional engineering, translational engineering tools have not yet been extensively developed. Although translational regulation in cellular systems is not as well-studied, these advances have shown to be effective in removing translation-level limitations.

### Post-translational engineering

Post-translational modification of proteins also takes place after translation and include phosphorylation, glycosylation, ubiquitination, methylation, acetylation, and proteolysis. Regulation of this process in the field of synthetic biology is especially important to either prolong or shorten the half-life of desirable proteins. To this end, addition of a synthetic ligand that binds to the destabilizing domains of specific proteins shields them from degradation, allowing fused proteins to perform their cellular functions in mammalian cells (Banaszynski et al., 2006). In addition, a synthetic gene network for tunable degradation of a tagged protein has been constructed in *S. cerevisiae* using components of the *E. coli* degradation machinery (Grilly et al., 2007), opening the door forengineering, and optimization of protein degradation for a variety of future applications in microbial cell factories.

### Pathway engineering

Once the enzymes are expressed from specific genes, the last major challenge lies in optimizing gene expression, protein abundance, enzyme activities, synthetic pathways, and metabolic products as a system, especially in a dynamic manner. To address this problem, researchers have recently developed an array of tools, including global regulator engineering (Hong et al., 2010), computational protein design (Samish et al., 2011), protein engineering (Bommarius et al., 2011), protein trafficking (Hou et al., 2012), protein scaffolds (Dueber et al., 2009), transporter engineering (Chen et al., 2013a), cellular efflux pump engineering (Dunlop et al., 2011), ultrasensitive input/output control system (Dueber et al., 2007), and computer-based complex gene circuits (Daniel et al., 2013). For example, transporter engineering through expression of heterologous ABC transporters from *Y. lipolytica* has been utilized successfully to significantly improve tolerance of *S. cerevisiae* against alkanes. In particular, the tolerance limit of *S. cerevisiae* against decane was increased about 80-fold (Chen et al., 2013a). Ultrasensitive switches with a non-linear input/output function can be effectively harnessed to control many complex biological behaviors in higher-order regulatory systems. These switches approximate digital behavior, providing an input detection threshold at which small changes in input concentration lead to large changes in output behavior. Another successful example of pathway engineering is computer-based complex gene circuits. Synthetic analog gene circuits were engineered to execute sophisticated computational functions in living cells using three transcription factors. Such circuits could lead to new applications for synthetic biology and biotechnology that require complex computations with limited parts (Daniel et al., 2013). These methods and technologies can be combined to optimize the metabolic pathway and significantly boost the production of target compounds in a controllable, scalable, and effective way within host cells.

## Conclusion and Future Perspectives

Fatty acid-derived diverse valuable chemicals are in great demand. This class of chemicals has recently been successfully produced by introducing different biosynthesis genes, enzymes, and pathways into various microbial hosts. Although much progress has been made in the use of metabolic engineering of microbes for the production of fatty acid-derived chemicals, the sub-optimal product yields, and productivities render these platforms far from reaching large-scale commercial exploitation.

Conventional metabolic engineering efforts on the microbial production of fatty acid-derived chemicals predominantly rely on identifying the activity of related enzymes isolated from different sources. In this regard, future efforts should be invested in finding and adopting novel sources of enzymes either in existing pathways or from completely novel producing pathways with such desired features as higher enzyme activity, stability, and specificity. High-throughput enzyme screening methods and bioinformatics tools could be used to screen these enzymes from vastly different organisms.

However, many attempts have demonstrated that the simple import of heterologous pathways into microbial hosts without a good understanding of complex regulatory networks underlying their biosynthesis pathways, will unlikely yield high-level production of target fatty acid-derived chemicals. Hence, the exploration of such metabolic and regulatory information is crucial for the heterologous production of these chemicals. Due to the complexity of regulatory networks, difficulties can be formidable. Synthetic biology-based tools can help to elucidate complex regulatory networks, enhance gene expression, increase enzyme activities and substrate specificity, improve metabolic flux, and boost product titer in heterologous microbial hosts. Taken together, combinatorial approaches encompassing metabolic engineering and synthetic biology together with more detailed knowledge of metabolic and genetic regulatory mechanisms, will be effective in overcoming bottlenecks inherent in the production of fatty acid-derived valuable chemicals in microbes. Ultimately, successful engineering strategies will be key to push efficient microbial-based production of the fatty acid-derived valuable chemicals forward toward industrialization.

## Conflict of Interest Statement

The authors declare that the research was conducted in the absence of any commercial or financial relationships that could be construed as a potential conflict of interest.
